# A Novel Mutation in KCDT7 Gene in an Indian Girl With Progressive Myoclonus Epilepsy

**DOI:** 10.7759/cureus.13447

**Published:** 2021-02-19

**Authors:** Sai Chandar Dudipala, Prashanthi M, Amith Kumar Chennadi

**Affiliations:** 1 Pediatric Neurology, Star Women & Children Hospital, Karimnagar, IND; 2 Pediatrics, Prathima Institute of Medical Sciences, Karimnagar, IND

**Keywords:** progressive myoclonus epilepsy, indian population, kcdt7 gene

## Abstract

The progressive myoclonus epilepsy (PME) is a rare group of clinically and genetically heterogeneous disorders characterized by myoclonus, drug refractory epilepsy, and neurological deterioration. Here, we report a three-year-old female patient with neuroregression after a period of normal development and uncontrollable myoclonic seizures, which fulfill the criteria of PME. Next-generation sequencing revealed a novel homozygous mutation of variant c.173G>C in exon 2 of the KCDT7 (potassium channel tetramerization domain containing protein 7) gene that was compatible with the diagnosis of progressive myoclonic epilepsy 3 (PME3) with or without intracellular inclusions. This is a rare report of KCTD7 mutations causing PME in the Indian population. Our findings supported the important role of KCTD7 in PME and broadened the mutation spectrum.

## Introduction

The progressive myoclonus epilepsy (PME) consists of myoclonic seizures, tonic-clonic seizures, progressive neurologic deterioration, resistance to treatment, and poor prognosis. These are rarely inherited neurodegenerative disorders due to various etiologies [1]. Myoclonus in PME is classically fragmentary and multifocal and often is precipitated by fever or external stimuli such as light or sound [2]. There are a large number of causes of the PME, most of which are due to specific genetic mutations [3]. To date, more than 30 known candidate genes for PME have been recognized.

Here, we report a three-year old Indian girl with PME, in whom next-generation sequencing led to the identification of a homozygous KCTD7 (potassium channel tetramerization domain containing protein 7) mutation. This is a rare report associating KCDT7 mutation with PME in the Indian population.

## Case presentation

The patient, a three-year-old Indian girl, was born of third-degree consanguineous Indian parents presented to the pediatric neurology clinic with complaints of seizures and regression of milestones. Pregnancy and birth history were uneventful with no indication of intrauterine or perinatal hypoxia or other neonatal complications. Seizures started when the patient was 10 months old after a period of normal development. When the patient was 10 months old, she was able to stand without support, transfer objects between hands, and wave bye-bye. The seizure types were continuous multifocal myoclonic seizures, affecting all the limbs and the face, and rare tonic and tonic-clonic seizures. She lost the ability to stand and transfer objects within two months of the onset of seizures. Neurological examination revealed irritability, central hypotonia, and myoclonus with a head circumference of 48 cm. There was no other significant abnormal finding on physical and other systemic examinations.

Electroencephalogram (EEG) showed multifocal epileptiform abnormalities. Magnetic resonance imaging (MRI) of the brain was normal at this time. On the basis of history, examination findings, and investigations, progressive myoclonic epilepsy due to genetic origin was considered. The patient was started on valproic acid, pyridoxine, and clobazam, but there was no response. Hence prednisolone was added. After these four medications, seizure frequency decreased. At 18 months of age, she was hospitalized with myoclonic status epilepticus, which was precipitated by pneumonia; at that moment the patient was treated with levetiracetam and clonazepam. After discharge from the hospital, the patient had increased frequency of seizures and neuroregression. When she was three years old, she was unable to hold her neck and recognize her parents. The patient is treated with several anti-seizure drugs like phenobarbital, valproic acid, zonisamide, topiramate, levetiracetam, clonazepam, and pyridoxine till today, but there is no improvement in the seizure frequency.

**Figure 1:**
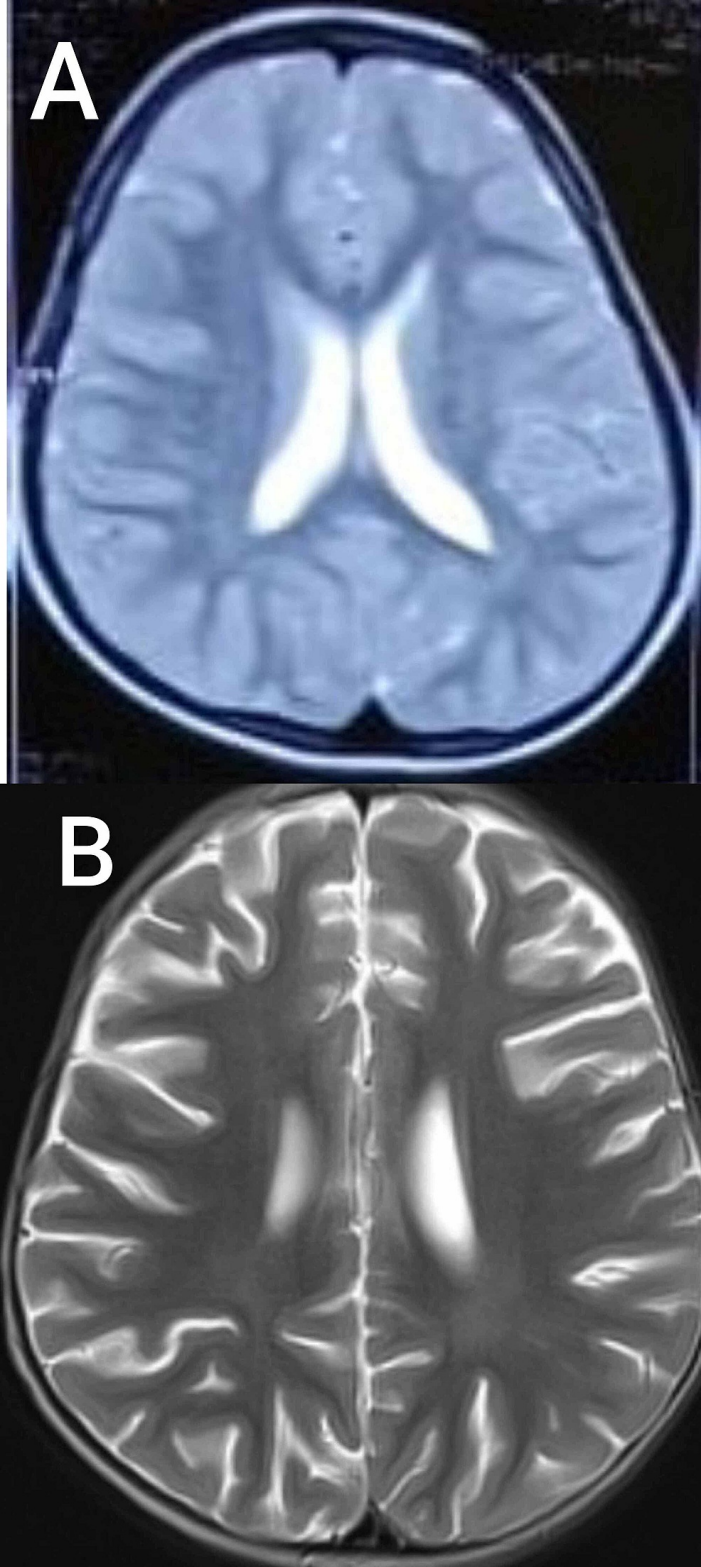
Magnetic resonance imaging (MRI) of the patient's brain (A) Axial T2-weighted image showing normal findings when the patient was one year old. (B) MRI of the brain showing diffuse atrophy when the patient was three years old.

Extensive investigations including complete blood picture, blood sugar, renal function tests, thyroid profile, lactate, vitamin B12, and vitamin D3 were normal. Tandem mass spectrometry was normal. Fundoscopy was normal. Cerebrospinal fluid analysis showed normal levels of glucose, proteins, lactate, and pyruvate. Karyotype showed the normal female type (46XX). When the patient was three years old, MRI of the brain showed generalized atrophy without any focal lesions (Figure 1).

After all the diagnostic work-up, on the ground of uncontrollable seizures, neuroregression, and consanguineous parents, genetic etiology was considered. DNA sequencing with next-generation sequencing platform revealed a pathogenic homozygous KCDT7 gene mutation, which causes an autosomal recessive progressive myoclonic epilepsy 3 (PME3). Previously this variant was rarely reported in the literature. Family screening and genetic counseling were advised.

## Discussion

PME is caused by a wide range of specific etiologies including neuronal ceroid lipofuscinoses, Unverricht-Lundborg disease, Lafora disease, myoclonic epilepsy with ragged red fiber, Tay-Sachs disease, sialidosis, and dentatorubral-pallidoluysian atrophy [2]. Most PMEs are due to various gene mutations: one of them is KCDT7 gene, and majorities are autosomal recessive [3]. The KCDT7 gene is located at 7q11.21. It encodes a member of the potassium channel tetramerization domain containing protein 7 family [4]. In 2007, Van Bogaert et al. reported homozygous mutation in KCDT7 gene in three members of a Moroccan family [5]. Since this report, 41 patients from 32 families have been reported [4-12]. Majorities were homozygous mutations, but three compound heterozygous mutations were also reported [6,9,13]. Kousi et al. reported various mutation in KCDT7 gene in eight patients from Turkish and Pakistani families [6]. In the present case, DNA analysis with next-generation sequencing showed a novel mutation of variant c.173G>C, pGly58Ala in exon 2 of the KCDT7 gene in a homozygous state, which is constant with PME3 with or without intracellular inclusions. We found this rare mutation related to PME in KCDT7 gene in the Indian population.

The exact molecular function of KCTD7 gene remains unknown. KCDT7 genes are expressed predominantly in the brain, specifically in cortical neurons, in granular and pyramidal cell layers of the hippocampus, and in cerebellar Purkinje cells. KCDT7 gene overexpression leads to hyperpolarization of the neuronal cell membrane and decreased neuronal excitability. Hence, loss of KCTD7 function may lead to depolarization of resting membrane potential and increased excitability with consequent susceptibility to epileptic seizures [14].

The individuals with KCDT7-related PME had a variable ethnic origin, high rate of consanguinity, and variable phenotypic presentation [5]. Age of onset ranged between five months to three years, after a period of normal or mildly delayed development. In the present study, onset of seizures was at 10 months of age. Epileptic seizures were the first manifestation in all reported cases. First seizures were described as either myoclonic or generalized tonic clonic seizures (GTCS) and were precipitated by fever in some cases. In the present report, the child had myoclonic seizures as initial seizures and had GTCS type also, and the child had myoclonic status epilepticus at 18 months of age along with pneumonia. This epilepsy is drug-resistant and has poor response to treatment in 2/3 of the patients. All patients had neuroregression at varying ages. In this study, the patient lost all her attained milestones at three years of age [5].

EEGs were abnormal in all the patients, and the most common findings were very frequent multifocal and/or generalized spike and waves. Our patient’s EEG findings were also consistent with this. MRI brain was either normal or non-specific; findings include atrophy or white matter changes. In the present case, first MRI brain was normal, and the next MRI brain done when the patient was three years old showed diffuse atrophy.

The phenotype of present case fulfills the criteria of PME with multifocal myoclonus, neuroregression after a period of normal development, multifocal spikes on EEG, and non-specific neuroimaging findings.

## Conclusions

In conclusion, next-generation sequencing will help to identify the cause of drug-resistant epilepsy with neuroregression in children. In this study, we have reported a rare case of KCDT7-related PME in the Indian population and identified a novel KCDT7 mutation in a child with PME. This study contributes to the genetic diagnosis and counseling of families with PME.
